# The response to background motion: Characteristics of a movement stabilization mechanism

**DOI:** 10.1167/jov.21.11.3

**Published:** 2021-10-07

**Authors:** Emily M. Crowe, Jeroen B. J. Smeets, Eli Brenner

**Affiliations:** 1 Department of Human Movement Sciences, Institute of Brain and Behavior Amsterdam, Amsterdam Movement Sciences, Vrije Universiteit Amsterdam, The Netherlands; 2 Department of Human Movement Sciences, Institute of Brain and Behavior Amsterdam, Amsterdam Movement Sciences, Vrije Universiteit Amsterdam, The Netherlands; 3 Department of Human Movement Sciences, Institute of Brain and Behavior Amsterdam, Amsterdam Movement Sciences, Vrije Universiteit Amsterdam, The Netherlands

**Keywords:** motor control, online control, visual guidance, background perturbation, movement stabilization

## Abstract

When making goal-directed movements toward a target, our hand deviates from its path in the direction of sudden background motion. We propose that this manual following response arises because ongoing movements are constantly guided toward the planned movement endpoint. Such guidance is needed to compensate for modest, unexpected self-motion. Our proposal is that the compensation for such self-motion does not involve a sophisticated analysis of the global optic flow. Instead, we propose that any motion in the vicinity of the planned endpoint is attributed to the endpoint's egocentric position having shifted in the direction of the motion. The ongoing movement is then stabilized relative to the shifted endpoint. In six experiments, we investigate what aspects of motion determine this shift of planned endpoint. We asked participants to intercept a moving target when it reached a certain area. During the target's motion, background structures briefly moved either leftward or rightward. Participants’ hands responded to background motion even when each background structure was only briefly visible or when the vast majority of background structures remained static. The response was not restricted to motion along the target's path but was most sensitive to motion close to where the target was to be hit, both in the visual field and in depth. In this way, a movement stabilization mechanism provides a comprehensive explanation of many aspects of the manual following response.

## Introduction

To successfully execute goal-directed movements in a constantly changing environment, we continuously use and integrate numerous sources of visual information (e.g., Brenner, Smeets, & de Lussanet, 1998; van der Kamp, Savelsbergh, & Smeets, 1997; de la Malla and López-Moliner, 2015; Brenner & Smeets, 2018). We make quick online adjustments to changes in a target's position (e.g., Georgopoulos et al., 1981; Goodale, Pelisson, & Prablanc, 1986; Prablanc & Martin, 1992; Brenner & Smeets, 1997; Day & Lyon, 2000; Gritsenko, Yakovenko, & Kalaska, 2009; Smeets, Oostwoud Wijdenes, & Brenner, 2016). We also respond to abrupt background motion, with our hand deviating from its path in the direction of such motion from approximately 150 ms after its onset (Brenner & Smeets, 1997; Saijo, Murakami, Nishida, & Gomi, 2005; Gomi, Abekawa, & Nishida, 2006). This manual following response (MFR) is very robust: It does not depend on the observer's actual postural stability (de Dieuleveult, Brouwer, Siemonsma, Van Erp, & Brenner, 2018) and is found in movements toward both visible objects (e.g., Mohrmann-Lendla & Fleischer, 1991; Brenner & Smeets, 1997) and memorized positions (e.g., Whitney, Westwood, & Goodale, 2003; Saijo et al., 2005; Gomi, Abekawa, & Shimojo, 2013). Why does the hand follow motion in the background?

The most compelling explanation for the MFR is that it is a response to inferring self-motion (Brenner & Smeets, 1997; Gomi, 2008). Specifically, an observer infers self-motion in the direction opposite to background motion. The hand then compensates for the estimated self-motion by moving in the opposite direction (i.e., in the direction of the background motion). One mechanism that could underlie the estimation of self-motion is an analysis of the instantaneous global optic flow: the structured patterns of retinal motion that result when an observer moves through the world (Gibson, 1950). Analysis of the global optic flow is believed to underlie many aspects of everyday behavior, including the visual guidance of locomotion (e.g., Gibson, 1958), gaze stabilization (e.g., Busettini, Masson, & Miles, 1997), updating perceived direction (e.g., Lepecq, Jouen, & Dubon, 1993), and maintaining posture (Lee & Aronson, 1974). More recently, Warren and Rushton (2008, 2009) demonstrated that observers separate retinal motion signals into components caused by observer movement and components caused by the movement of objects in the scene and that doing so involves a global analysis of retinal motion (Warren & Rushton, 2007). Estimating self-motion according to the instantaneous global optic flow therefore seems a plausible mechanism that might underlie the MFR.

However, various experimental characteristics of the MFR are inconsistent with the proposal that self-motion is estimated on the basis of the instantaneous global optic flow. First, the MFR is primarily based on background motion close to the target (Brenner & Smeets, 2015) or where one is looking (Abekawa & Gomi, 2010). This suggests that task-relevant regions of the display are particularly important, which is inconsistent with evidence that the effectiveness of estimating self-motion from the instantaneous global optic flow is uniform across the visual field or maybe even higher in the peripheral visual field (Palmisano & Gillam, 1998; Raffi & Piras, 2019). Second, the MFR is more prominent than postural responses to background motion (Zhang, Brenner, Duysens, Verschueren, & Smeets, 2018), suggesting that the MFR is not a compensation for the same estimated self-motion that leads to the observed postural response. Finally, galvanic and visual stimulation that lead to a similar postural response (presumably compensating for similar estimates of self-motion) lead to MFRs that differ considerably (Zhang, Brenner, Duysens, Verschueren, & Smeets, 2019). Since the response to inferred self-motion should be independent of the sensory modality that gives rise to the estimate of self-motion, one would not expect this difference in the magnitude of the MFR. Together, these experiments suggest that the MFR is not the result of estimating self-motion on the basis of an analysis of the global optic flow and compensating accordingly.

An alternative idea is that the MFR is the result of updating the endpoint of the movement to match any motion near that endpoint because any such motion might indicate that the egocentric position of that endpoint has changed due to self-motion. Such a mechanism would involve selecting a region to rely on and responding to any motion in that region rather than being based on an estimate of the global optic flow. In six experiments, we specify the details of this idea and refine our estimate of the region that such a mechanism considers. Experiments 1 to 3 confirm that any motion near the movement endpoint is sufficient to elicit an MFR. The response does not consider the background's stability (Experiment 1) and occurs even when the majority of background items are static (Experiments 2 and 3). Experiments 4 to 6 characterize the spatial constraints of this mechanism and show that location matters in terms of proximity to the movement endpoint, with a bias toward the lower visual field. These results support the idea that the MFR is the result of a *movement stabilization mechanism* whereby the planned endpoint of one's action is shifted in the direction of any motion in its vicinity. This mechanism presumably helps the quick stabilization of ongoing movements when there is not very precise or reliable information about self-motion with respect to the planned endpoint.

## Methods

**Figure 1.**
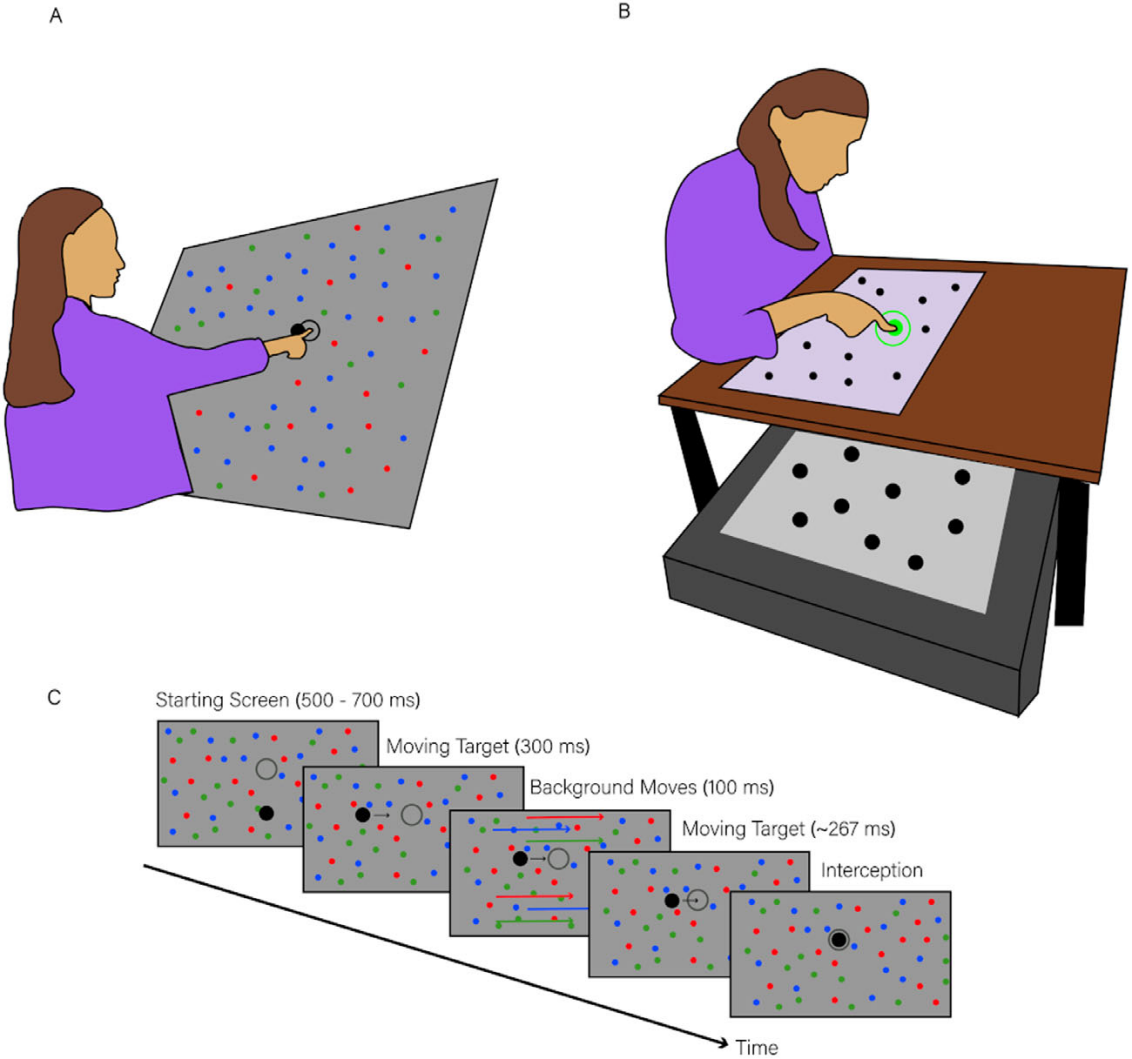
Details of the task. (A) A schematic representation of the setup for Experiments 1 to 5, with the participant standing in front of a slanted screen on which the stimuli were displayed. (B) A schematic representation of the setup for Experiment 6 with the participant standing in front of two horizontal screens in a condition in which the target was on the near screen. (C) The timeline of the task. The times given in brackets denote the duration of each part of the task, and the images reflect the display from Experiments 1 and 2.

To be able to distinguish between the visual target and the planned movement endpoint, we used moving targets that participants had to intercept. Participants stood in front of a large screen. They kept their index finger on a starting position until a target appeared moving to the right (Figures 1A and 1B). We asked participants to intercept this target when it was within a static interception zone. It was within this zone from 567 to 767 ms after it appeared. Having an interception zone restrained both the time and location of the interception. We did not constrain the participant in any other way because this most closely captures what happens in natural situations. Previous research showed that the hand responded to background motion after approximately 150 ms and continued to follow the background motion for around 100 ms (Brenner & Smeets, 1997; Gomi et al., 2006; Brenner & Smeets, 2015). We chose to start moving the background 300 ms after the target appeared to be sure to capture the hand's maximum deviation from its standard trajectory, even if the target was hit before it reached the center of the interception zone. The background motion could either be leftward or rightward. By taking the differences of the responses to these two perturbations, we get rid of any bias in any movement strategies, so that a pure response to background motion remains.

### Participants

Each of the six experiments was performed by 12 participants. Several participants completed more than one of the experiments (Table 1). Participants 23 to 42 participated in return for course credit, and all other participants volunteered. Participants were not aware of the manipulations under study, but most of the manipulations were quite evident. This study was part of a research program that has been approved by the local ethics committee in accordance with the Declaration of Helsinki. All participants gave written informed consent.

**Table 1.** Participant demographics for each experiment. Age is in years (with the standard deviation across participants). Handedness as reported by the participant.

Experiment	*n*	Age (years)	Gender	Handed	Participant IDs
1	12	28 ± 3	5 female	12 right	1–12
2	12	27 ± 3	7 female	11 right	3, 8, 13–22
3	12	23 ± 4	8 female	11 right	2–3, 23–32
4	12	27 ± 4	8 female	10 right	4, 13, 33–42
5	12	21 ± 3	9 female	12 right	10, 14, 17, 19, 43–50
6	12	27 ± 3	8 female	11 right	1–3, 8, 12, 14, 51–56

### Setup

Experiments 1 to 5 were conducted in a normally illuminated room. The stimuli were presented at 120 Hz (InFocus DepthQ Projector (Maxnerva Technology Services Limited, Kowloon, Hong Kong); resolution: 800 by 600 pixels). They were projected from behind onto a 1.00-m by 1.25-m (height by width) acrylic rear-projection screen (Techplex 150; Stewart Filmscreen Corporation, Torrance, CA, USA) that was tilted backward by 30⁰ (Figure 1A). Participants stood in front of the screen and tapped the screen with their dominant index finger. They were not restrained in any way. An infrared camera (Optotrak 3020; Northern Digital, Waterloo, ON, Canada) that was placed at about shoulder height to the left of the screen measured the position of a marker (an infrared light-emitting diode) attached to the nail of the participant's dominant index finger at 500 Hz. In order to synchronize the movement data (i.e., the marker position) with the stimulus presentation, the camera also recorded the position of a second marker attached to the side of the screen. This marker did not move, but it stopped emitting infrared light so that its position was registered as “missing” when a flash was presented at the top-left corner of the screen (where a light sensor was placed to detect the flash).

Experiment 6 was conducted in a dark room to compensate for the lower luminance of the setup. The stimuli were presented on two horizontal screens (refresh rate of 60 Hz; resolution of 1,920 by 1,080 pixels), separated vertically by 20 cm (Figure 1B) to allow us to present images at two distances. We refer to the top and bottom screen as near and far, respectively, to indicate their distance from the participants’ eyes. The far images were presented on the bottom screen (image size 92 by 52 cm) that had a white background. The near images were presented on the top screen (60 by 33 cm) that was embedded in a large (70 by 70 cm) surface that rested on four 25-cm-high columns. These columns ensured that the participants were able to move their arm freely below the top screen. The top screen was a standard monitor that had been dismantled: The background and lighting were removed so that it blocked vision when it was set to black and participants could see through it when it was set to “white.” Participants directed their gaze downward toward the screens, with their eyes approximately twice as high above the top screen than above the bottom screen. There was a light sensor in the top-right corner of both screens, and a signal was sent to the movement data output when light fell on either sensor. We used the same methods as in Experiments 1 to 5 to collect movement data and synchronize these data with the stimulus presentation.

### Calibration

At the beginning of each session, the position of the marker on the fingertip was measured while the participant positioned the fingertip at four indicated positions on the screen. This simple 4-point calibration was used to relate the position of the fingertip to the projected images, automatically correcting for the fact that the marker was attached to the nail rather than the tip of the finger. For Experiment 6, this calibration was conducted on the far screen (the relative positions of pixels on the two screens were determined in advance).

### Stimulus and procedure

Participants could stand and move in front of the setups in any way they felt would help them perform the task. All measurements are therefore presented in centimeters rather than degrees of visual angle, because the latter differed between participants and trials. Participants started each trial by placing their index finger at a starting point and waited until a target appeared. If participants lifted their finger from the starting point before the target appeared, the target did not appear and they had to place their finger back at the starting point. Participants could rest whenever they wanted to by not placing their finger at the starting point.

In Experiments 1 to 5, the display consisted of a gray background with 600 dots with a 1-cm diameter scattered across it at random (there was a different random configuration on each trial). In Experiments 1 and 2, the background dots were red (200), green (200), and blue (200). In Experiments 3 to 5, all the background dots (600) were black. The starting point was a 4-cm diameter black disk that was 30 cm below the interception zone. The target was a 2-cm diameter disk, which was black except in Experiment 2, in which it could have one of three colors (red, green, or blue). The target first appeared 20 cm to the left of the screen center. In Experiments 1 to 3, it was displayed 10 cm above the screen center; in Experiment 4, it was displayed 5 cm above the screen center; and in Experiment 5, it was displayed at the screen center. The interception zone was a 6-cm diameter black ring presented at the horizontal center of the screen. The vertical position of the interception zone was identical to that of the target.

In Experiment 6, the displays consisted of white backgrounds with 200 black dots with a diameter of 0.5 cm on the near screen and 118 black dots with a diameter of 1 cm on the far screen. These numbers and dimensions were chosen to approximately match the angular density and size of the dots across the screens. The starting point was 20 cm from the interception zone. On both screens, both the interception zone and target were green and the starting point was black. Participants always moved their hand on the screen on which the starting point, target, and interception zone were presented. The sizes of the starting point, target, and interception zone were the same as in all other experiments.

After the finger had been at the starting point for a randomly chosen time between 0.5 and 0.7 s, the starting point disappeared and the target appeared (Figure 1C). The target moved to the right at 30 cm/s. Participants were instructed to tap the target when it was in the interception zone. The target reached the center of the interception zone 667 ms after it appeared. Since the task was to tap the target when it was in the interception zone, moving the background at a predefined time meant that it moved at an almost fixed time with respect to the expected time of the tap. Specifically, the background moved from 300 to 400 ms after the target appeared, which is between 367 and 267 ms before the anticipated time of the tap. The background moved at a constant speed of 20 cm/s (covering 2 cm during the 100 ms of background motion) except when the background moved on the far screen in Experiment 6, in which case, it moved at 40 cm/s (covering 4 cm in the 100 ms of motion). The motion was faster on the far screen so that the angular velocity was approximately the same on both screens.

In order to provide participants with feedback on their hitting performance, we detected taps online. A tap was detected if the reduction in the distance to the screen between consecutive measurements decreased by more than 1 mm (i.e., a deceleration threshold of 50 m/s^2^) while the finger was less than 2 cm above the screen. If the position of the fingertip (as determined during calibration) was within the outline of the target at the moment of the tap, we considered that target to have been hit. If a target was hit, it remained at the position at which it was hit for 500 ms. If the center of the target was within the interception zone when it was hit, there was a sound indicating that the hit was successful. If a target was missed, it deflected away from the finger at 100 cm/s, also remaining visible for 500 ms unless it left the screen before that. For example, if the finger tapped above and to the left of the target, the target moved down and to the right at 100 cm/s for up to 500 ms (after the tap) before disappearing. All the delays in our equipment were considered when determining the target's position at the moment of the tap. The position of the target was interpolated between image presentations.

### Data analysis

To evaluate the time course of the effects of the background motion, we first converted the measured lateral positions of the finger (i.e., parallel to the moving target) into (signed) lateral velocities by direct differentiation. This was done for every 2-ms interval for the first 300 ms after the onset of background motion (300–600 ms after the appearance of the moving target). For each participant, we then averaged the lateral velocity of the finger for every interval. We did so separately for trials in which the background moved leftward and ones in which it moved rightward. We subsequently determined the response by subtracting the average velocity for leftward background motion from that for rightward background motion. We did this for each condition of each experiment.

All trials containing background motion were included in the analysis of all experiments, irrespective of performance. After determining the individual responses in each condition, the values were averaged across participants. We present the time course of the responses to background motion for each experiment (mean values and standard errors across participants). We quantified the initial response of the hand for each participant and condition by taking the mean response between 150 ms (the time at which we anticipated the hand to start responding) and 200 ms after the background started moving. We used this initial response for our statistical analysis. We used either paired *t* tests or repeated-measures analyses of variance (ANOVAs) to assess whether differences in the response between conditions were consistent across participants. When it is not evident whether there was a response in a specific condition, we provide the 95% confidence intervals of the initial response for that condition.

## Experiments

### Experiment 1

Since surfaces in natural situations are largely static, retinal image motion that arises from background motion is often considered more likely to reflect self-motion than actual motion of the background (Gibson, 1979). However, attributing background motion to self-motion only makes sense if one expects the environment to be static. Are we indeed less likely to respond to background motion if there is visual evidence that the background is unstable? If any motion near the planned endpoint is sufficient to elicit the MFR, then the stability of the background should be irrelevant.

We introduced three conditions: high, medium, and low stability. As our aim was to change the assumptions about the stability of the background, we presented each of these three conditions in a separate block of 200 trials. The order of the blocks was counterbalanced across participants. In the medium-stability block, the background dots that appeared at new positions at the onset of each trial remained static until they started to move coherently for 100 ms (100 leftward; 100 rightward). The high-stability block was similar, except that the background dots only moved on 20% of the trials (20 leftward; 20 rightward); the background remained static in the other 80% of the trials. In the low-stability block, the background dots had asynchronous limited lifetimes: Each dot was replaced by another dot at a random position on the screen every 300 ms. Thus, the background was constantly changing, which should raise doubts about its stability. In this condition, the background also moved leftward on 100 trials and rightward on 100 trials. Leftward, rightward, and no-motion (in the high-stability block) trials were all interleaved within a block. The entire experiment took approximately 40 min, including short breaks between the blocks.

#### Results and discussion

**Figure 2.**
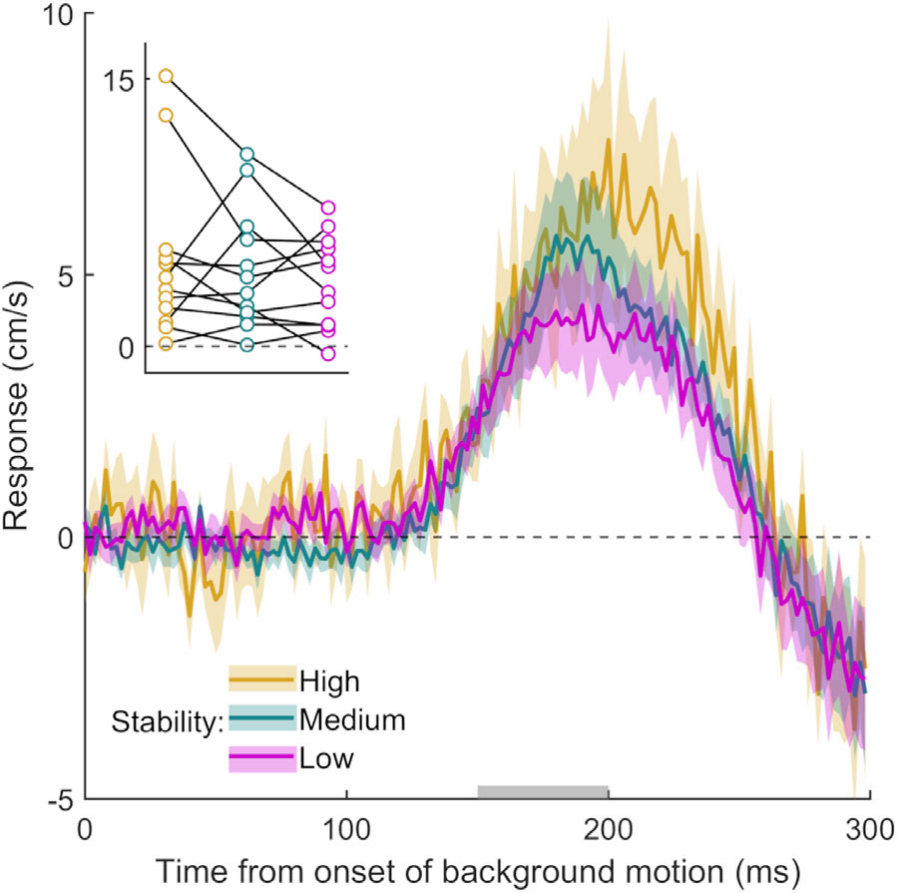
Time course of the response to background motion in Experiment 1. Each curve shows the difference between the mean horizontal hand velocity on leftward and rightward trials, averaged across participants. Shaded regions show the standard error of the mean across participants. A positive response is in the direction of background motion. The inset shows each participant's initial response to background motion (the average value between 150 and 200 ms from the onset of background motion; time interval indicated in gray) in each condition. Individual participants’ data points are connected by lines. The experiment consisted of three blocks that differed in the stability of the background. The colors of the curves and data points represent the three levels of stability.

Participants hit the screen 673 ± 20 ms (here and elsewhere, values are presented as means ± standard deviations across participants’ mean values) after the target appeared and hit the target within the interception zone on 84 ± 13% of the 600 trials. The participants’ hands responded within 150 ms to the onset of background motion in all three stability blocks (response curves deviate from zero in Figure 2). The latency appears to be slightly less than the 150 ms that has been reported previously (e.g., Brenner & Smeets, 2015). Most important, even when the background consisted of limited lifetime dots and was clearly not stable (purple curve), the hand was still pulled in the direction of background motion. The initial responses look extremely similar despite the large differences in background stability (see Figure 2, inset). A repeated-measures ANOVA did not reveal a significant effect of background stability on the magnitude of the MFR, *F*(2, 22) = 0.81, *p* = 0.46. This shows that the preceding stability of the environment does not modulate the MFR substantially and systematically. Finding a clear MFR irrespective of whether the background moved on every trial or only occasionally, as well as whether the pattern of the background itself was constantly changing, fits with the idea that we outlined at the end of the Introduction: Any motion close to the planned endpoint of the action elicits a response.

### Experiment 2

Backgrounds typically consist of multiple structures, but the majority of research to date used backgrounds consisting of one relatively large textured structure (e.g., Whitney et al., 2003; Gomi et al., 2013; Saijo et al., 2005; Kadota & Gomi, 2010). Two studies have used backgrounds consisting of multiple structures to investigate experimental characteristics of the MFR. Using a display of independent squares that had a limited lifetime, Saijo et al. (2005, Experiment 3) showed that reducing the coherence of the squares’ motion reduced the magnitude of the MFR. Brenner and Smeets (2015) showed that not all of the independent squares composing a checkerboard display had to move for the MFR to emerge. In that study, the location of the background structures that moved was random, but these structures were relatively large, making it difficult to assess the role of static independent structures within the same region near the movement endpoint. Is the MFR still present when the majority of background elements remain static? If any motion is sufficient to lead to an updated planned endpoint, the MFR should be observed even if only a small portion of background elements move.

Moreover, if *any* motion is considered, features other than spatial location should not modulate the MFR. We therefore examined whether people are more likely to respond to the motion of background structures that are similar to the target. Since color processing takes place early in the visual system (Gegenfurtner & Kiper, 2003) and color can contribute to the fast adjustment of ongoing arm movements (Brenner & Smeets, 2004), we investigated whether the similarity in color between the target and moving items in the background modulates the MFR.

Participants completed three blocks of 200 trials, one for each target color (red, green, and blue), the order of which was counterbalanced across participants. At the fixed time, 200 of the 600 dots moved horizontally: those of one of the three colors. In the color-congruent condition (100 trials: 50 leftward and 50 rightward), the background dots that moved had the same color as the target (e.g., both the target and the background dots that moved were red). In the 100 trials of the color-incongruent condition, the background dots had a color that differed from the target (e.g., the target was red and either the blue or the green background dots moved; 50 trials each: 25 leftward and 25 rightward). The trials of the two conditions were interleaved within each block. The entire experiment took approximately 40 min, including short breaks between blocks.

#### Results and discussion

**Figure 3.**
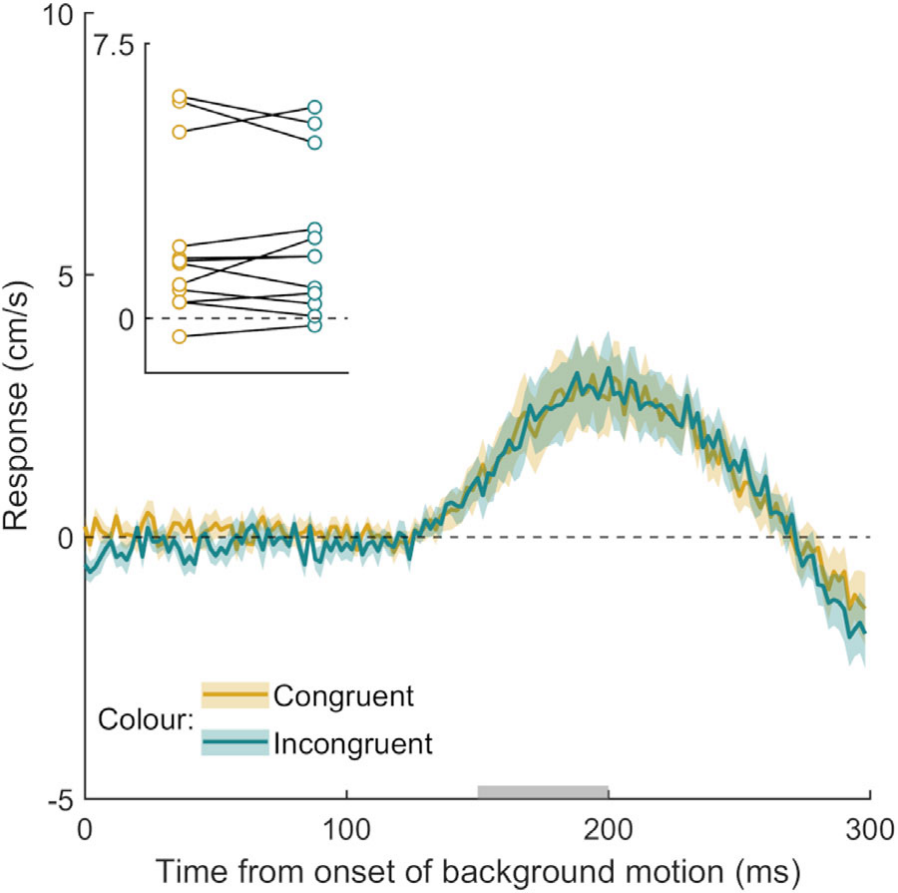
Time course of the response to background motion in Experiment 2. In this experiment, only one third of the background items moved. The gold curve shows the response when the color of the moving items in the background was congruent with that of the target. The turquoise curve shows the response when their color was incongruent (i.e., when the moving background items have one of the other two colors). Further figure details are the same as Figure 2.

Participants hit the screen 676 ± 14 ms after the target appeared and hit the target within the interception zone on 87  ± 7% of the 600 trials. Despite the fact that twice as many dots remained static than moved, there was a clear MFR. This suggests that static dots are ignored and any abrupt motion is sufficient to drive the MFR, rather than the MFR being the consequence of estimating self-motion based on the overall motion signal present in the display (Figure 3). A paired samples *t* test showed no evidence for a difference in the magnitude of the MFR in the color-congruent and color-incongruent conditions, *t*(11) = 0.07, *p* = 0.949. Thus, the fact that the target had a certain color did not give motion of background structures that had the same color more influence.

### Experiment 3

Experiment 2 showed that the MFR was still present when most background structures remain static. Are there a minimal number of background structures that must move to elicit the MFR? Since the MFR is primarily driven by motion near the movement endpoint (Brenner & Smeets, 2015), we moved background structures that were either near the target or near the interception zone. On each trial, only three of the 600 background dots (0.5%) moved horizontally for 100 ms. Participants completed 400 trials. On 200 trials, the three moving background dots were at random positions within a 6-cm × 6-cm box centered on the interception zone. On the other 200 trials, the three dots that moved were within a similar box centered on the target's position during the background motion. In each case, the dots moved to the left on half the trials and to the right on the other half. All these conditions were interleaved in an experiment that lasted approximately 30 min.

#### Results and discussion

**Figure 4.**
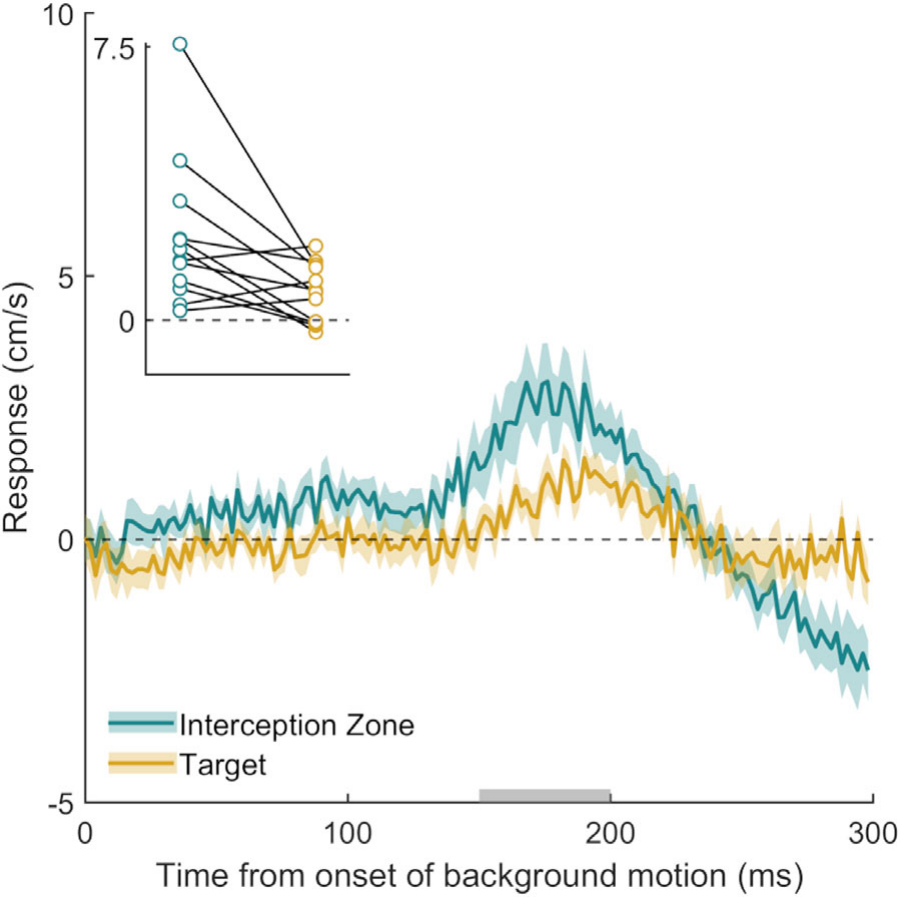
Time course of the response to background motion in Experiment 3. The gold curve shows the response when background motion was at the target location. The turquoise curve shows the response when the background motion was at the interception zone. Further figure details are the same as Figure 2.

Participants hit the screen 675 ± 10 ms after the target appeared and hit the target on 82  ± 8% of the 400 trials. Most important, the participants responded to the motion of the three dots in the background (Figure 4). The response was present at both locations; for the initial response at the target location, the 95% confidence interval was 0.27 to 1.28 cm/s. The response was stronger when the background dots moved near the interception zone than when they moved near the target, *t*(11) = 2.81, *p* = 0.017. The finding that the motion of only three dots surrounded by 597 static dots induces a clear response provides even stronger evidence than Experiment 2 that only the moving dots matter, irrespective of any evidence for a static background. The finding that motion at both locations induces a responses suggests that it is not a specific visual item that is affected.

The results from these first three experiments are inconsistent with the MFR being a compensation for estimated self-motion based on an instantaneous analysis of the global optic flow, because even when the background is not stable at motion onset (background dots are constantly changing position) and when self-motion would not be inferred from the instantaneous optic flow (most background dots are static), the MFR was still present. The clear response to three dots moving when they were near the interception zone supports the idea that motion near the movement endpoint is critical. The MFR is strongest in response to motion near the interception zone, but motion elsewhere is also relevant. We therefore investigated in Experiments 4 to 6 how the MFR depends on the spatial relations between background motion and planned endpoint.

### Experiment 4

The MFR is most sensitive to background motion in specific areas. These areas have been identified as being near one's gaze (Abekawa & Gomi, 2010) or near where the target and the interception zone are located (Brenner & Smeets, 2015). Does *only* motion that occurs within a few degrees of visual angle of the target's path contribute to the hand's response? If so, motion that occurs a considerable distance below the target's path should have no effect.

In line with the previous experiments, participants were asked to intercept a moving target when it reached the interception zone, but now the upper and lower halves of the background could move in opposite directions. If the MFR is a specific response to motion that occurs along the targets’ path, we would expect the response to follow the background motion in the upper half of the display, irrespective of the motion in the lower half. We therefore compared the influence of background motion in two conditions: one in which the two halves of the display moved in opposite directions and one in which they moved in the same direction. We placed the border between the regions with opposite motion just below the targets’ path (the border was 5 cm below the center of the target and interception zone; this generally corresponds with a distance of about 5° of visual angle). If only motion with a few degrees of visual angle of the target's path matters, only the motion of the upper half should matter, so the response should be in the direction of motion of the upper half, irrespective of how the lower half moves. Participants completed one block of 400 trials in which the dots in the two halves of the background either moved in the same direction (200 trials, 100 for each direction of motion) or moved in opposite directions (200 trials, 100 for each direction of motion in the upper half of the screen). These conditions were randomly interleaved. This experiment took approximately 30 min.

#### Results and discussion

**Figure 5.**
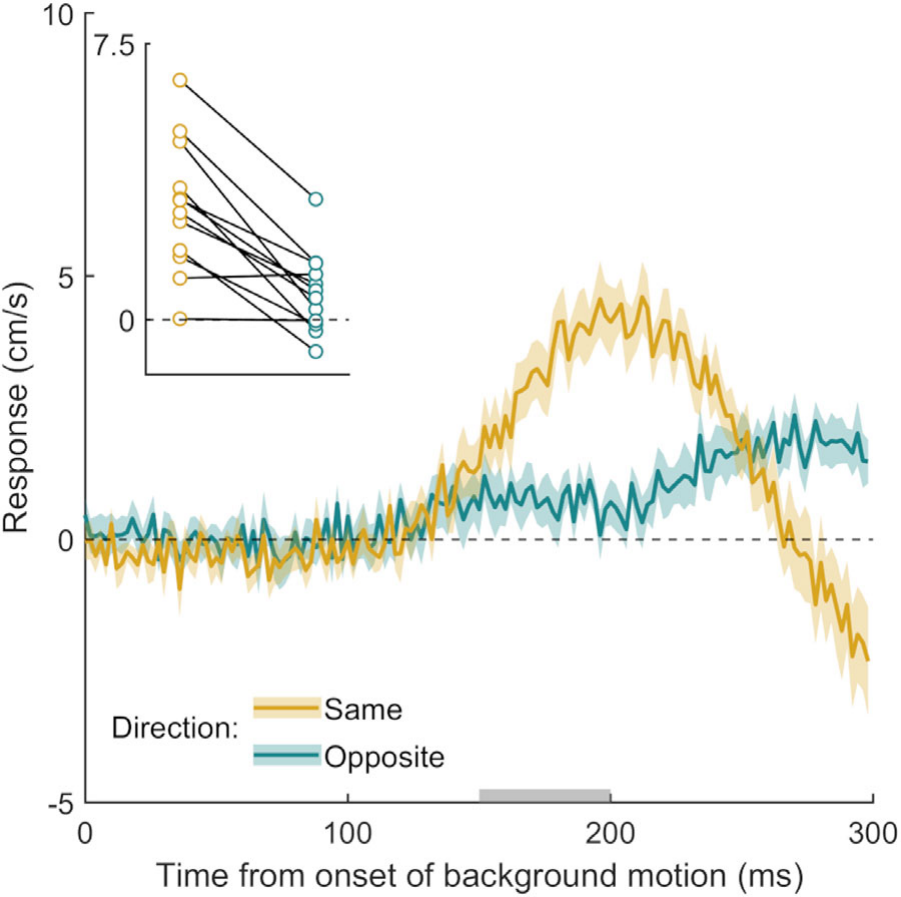
Time course of the response to background motion in Experiment 4. The background in the lower half of the screen could move in the same direction as that in the upper half of the screen (which included the interception zone; gold curve), or it could move in the opposite direction (turquoise curve). A response is positive if it is in the direction of the background motion in the upper half of the screen. Further figure details are the same as Figure 2.

Participants hit the screen 678 ± 17 ms after the target appeared and hit the target on 84  ± 12% of the 400 trials. In both conditions, we subtracted the response of the hand when the upper half moved leftward from its response when the upper half moved rightward. A positive MFR therefore reflects the hand following the direction of the background motion presented in the upper half of the screen. The usual response to background motion was present when all dots moved in the same direction (see Figure 5, gold curve). The MFR was much weaker when the two halves of the screen moved in opposite directions, *t*(11) = 5.70, *p* < 0.001.

When the two halves of the screen moved in opposite directions, the hand deviates in the direction of background motion in the upper half of the screen (the 95% confidence interval for the initial response is 0.04–1.43 cm/s; see Figure 5, turquoise curve). In this case, however, the MFR follows a qualitatively different pattern with a later peak response. Motion in the lower half of the screen is thus certainly not irrelevant, showing that not only motion along the targets’ path drives the MFR. Although finding some influence of motion in the lower half of the screen was not that surprising, given that the distance between the interception zone and the border of the lower half of the screen was only a few centimeters, we were surprised that the MFR was so much smaller. Danckert and Goodale (2001) found more effective visually guided pointing in the lower visual field. They proposed that this reflects a functional bias for processing visual cues in this region during the online control of action, which suggests that there may be a tendency to rely more on background motion presented below the interception zone.

### Experiment 5

Experiment 4 showed an unexpectedly large effect of background motion in the lower half of the screen. Is this due to a bias to respond to visual information presented in the lower visual field? If so, we would expect to see a stronger MFR in response to motion presented exclusively in the lower half of the screen compared with motion presented exclusively in the upper half of the screen. Participants typically track the target when performing this kind of interception task (Cámara, de la Malla, López-Moliner, & Brenner, 2018), but since we did not measure gaze, we report our results according to the display location (i.e., upper or lower half of the screen) in which the background dots moved. In this experiment, we moved the target and interception zone to the vertical center of the screen. We did this to ensure that the regions with dots above and below the center had the same size. We moved the starting point downward accordingly, such that the hand still traveled the same distance to perform the interception. Only background dots either above or below the center moved on a given trial. Participants completed 200 trials in the condition in which only the top half of the background moved and 200 trials in which only the bottom half of the background moved. In each condition, background motion was to the left in 100 trials and to the right in the other 100 trials. All these trials were interleaved, and the experiment took approximately 30 min.

#### Results and discussion

**Figure 6.**
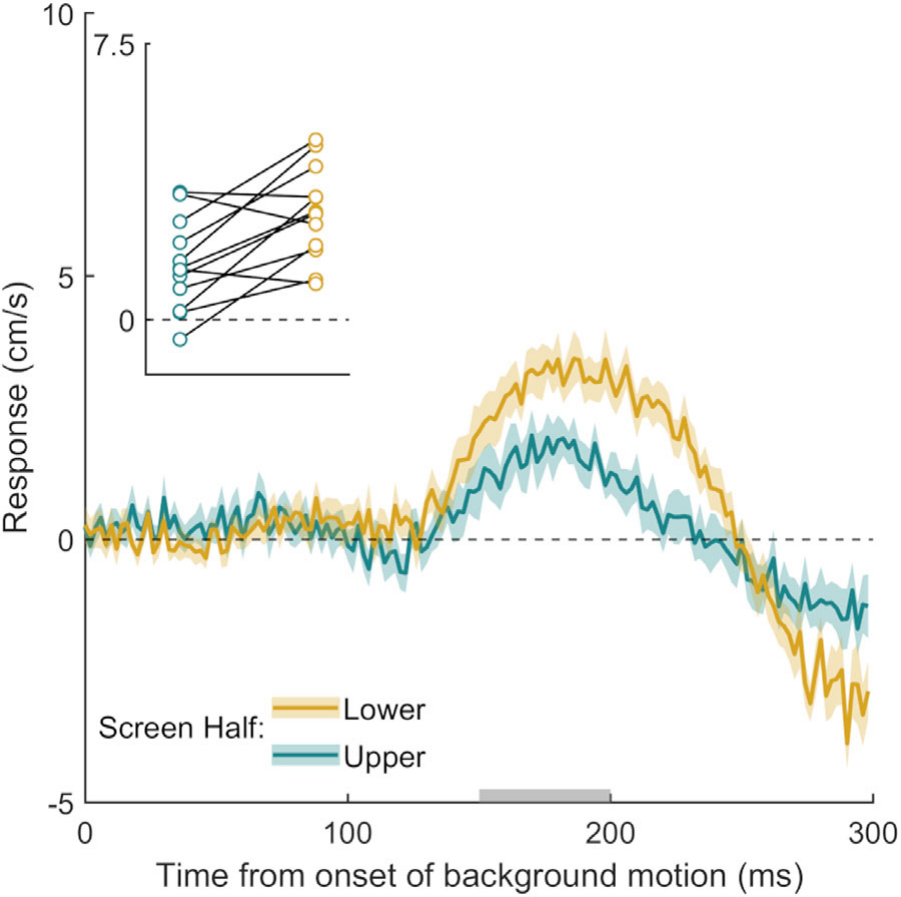
Time course of the response to background motion in Experiment 5. The gold curve shows the response to background motion in the lower half of the display, and the turquoise curve shows the response to background motion in the upper half of the display. Further figure details are the same as Figure 2.

Participants hit the screen 673 ± 20 ms after the target appeared and hit the target on 79  ± 10% of the 400 trials. The hand responded to background motion in both the upper and lower halves of the screen, but the magnitude of the MFR was clearly larger for motion in the lower part of the visual field (Figure 6). A paired samples *t* test confirmed that this difference was consistent across participants, *t*(11) = 3.69, *p* = 0.004.

This result is consistent with visual cues in the lower visual field being more effective at guiding ongoing actions (e.g., Danckert & Goodale, 2001), assuming participants directed their gaze toward the target (Cámara et al., 2018). The lower visual field may also be given more weight since this is the region from which the hand approached the target and from which the hand usually approaches targets that we fixate when reaching out for them (we normally fixate objects that we intend to grasp; e.g., Voudouris, Smeets, Fiehler, & Brenner, 2018).

### Experiment 6

There is evidence that the MFR depends on motion near where the target will be hit (Brenner & Smeets, 2015; Experiments 3 and 4). Is motion delineated on the retina or does depth also play a role? To examine this, we used a two-screen setup in which background dots were present on both screens (Figure 1B). Two experiments (6a and 6b) consisted of four randomly interleaved combinations of where the background motion and moving target were presented. Participants completed two blocks of 400 trials (Experiments 6a and 6b), which took approximately 1 hr.

In Experiment 6a, only the background dots on one screen moved. Does the magnitude of the MFR depend on where the target and background are presented relative to each other in depth? If the region within which background motion gives rise to a MFR is defined in actual space, we will find the largest response to background motion on the same screen as the target. If the region is defined on the retina, it should not matter whether the background motion and the target are on the same screen or on different screens. The experiment consisted of four conditions: all combinations of the two locations of the background motion (near or far) and relative location of target (same or different screen to the background). Experiment 6a consisted of 400 trials, 100 (50 leftward and 50 rightward) for each condition, randomly interleaved.

In Experiment 6b, the background dots moved on both screens. They either moved in the same direction on both screens or in opposite directions. Except for verifying whether the depth dimension is considered when selecting the region within which abrupt motion is followed with the hand, we were also interested whether we can interpret the effect of both backgrounds moving (Experiment 6b) as the sum of the effects of the individual backgrounds moving (Experiment 6a). This might be so if any abrupt motion influences the movement, irrespective of what other structures are doing. Experiment 6b consisted of 400 trials, 100 (50 leftward and 50 rightward) for each condition, randomly interleaved.

#### Results and discussion

**Figure 7.**
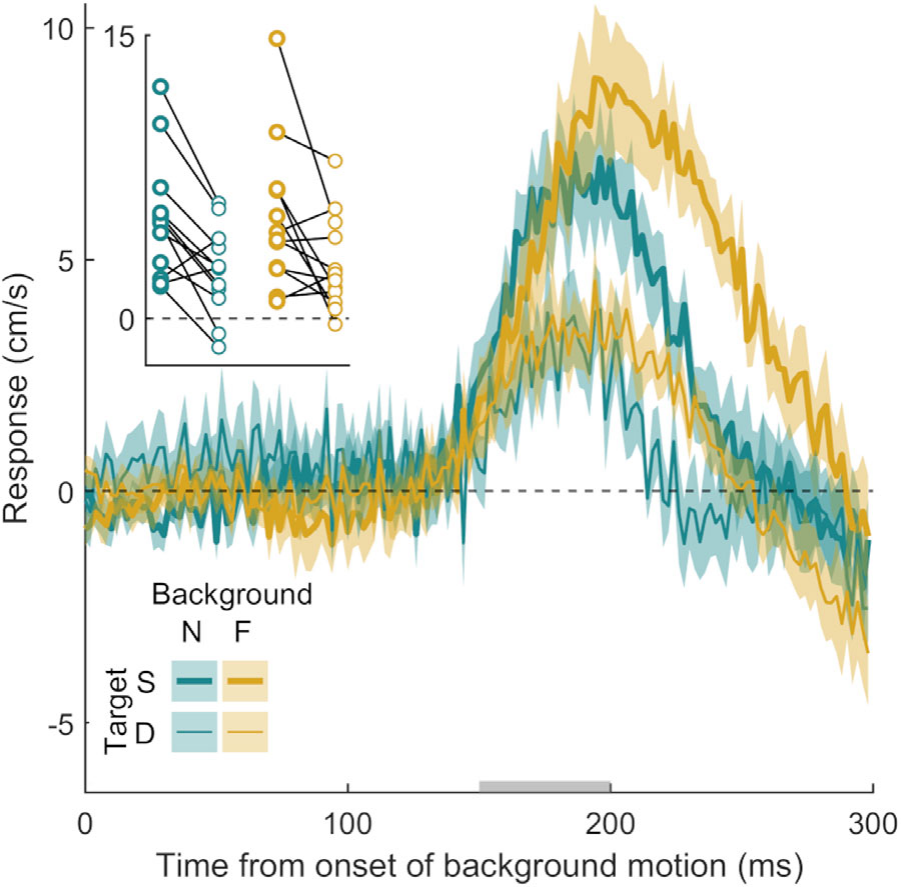
Time course of the response to background motion in Experiment 6a. The golden curves show the response to background motion on the far screen, and the turquoise curves show the response to background motion on the near screen. The thick curves correspond to the conditions in which the target and background motion were presented on the same screen; the thin curves correspond to the conditions in which they were presented on different screens. Further figure details are the same as Figure 2.

Participants hit the screen 666 ± 22 ms after the target appeared and hit the target on 67  ± 2% of the 400 trials. The hand deviated from its usual path in the direction of background motion in all four conditions (Figure 7). The hand's response was larger when the target was presented on the same screen as the background motion (Figure 7, thick curves) than when they were presented on different screens (thin curves), a difference that was consistent across participants (2 × 2 ANOVA: *F*(1, 11) = 11.43, *p* = 0.006, and inset of Figure 7). This shows that background motion gives rise to a stronger MFR if it is presented at the same depth as where the target will be hit. The initial response of the hand was similar when the background motion was on the far screen (Figure 7, gold curves) as when it was on the near screen (Figure 7, turquoise curves), *F*(1, 11) = 0.07, *p* = 0.792, suggesting that the depth itself did not matter. There was no interaction between the location of background motion and whether or not the target and background motion were on the same screen, *F*(1, 11) = 0.22, *p* = 0.645. Later parts of the response clearly depend on whether the background motion was presented on the near compared with the far screen.

**Figure 8.**
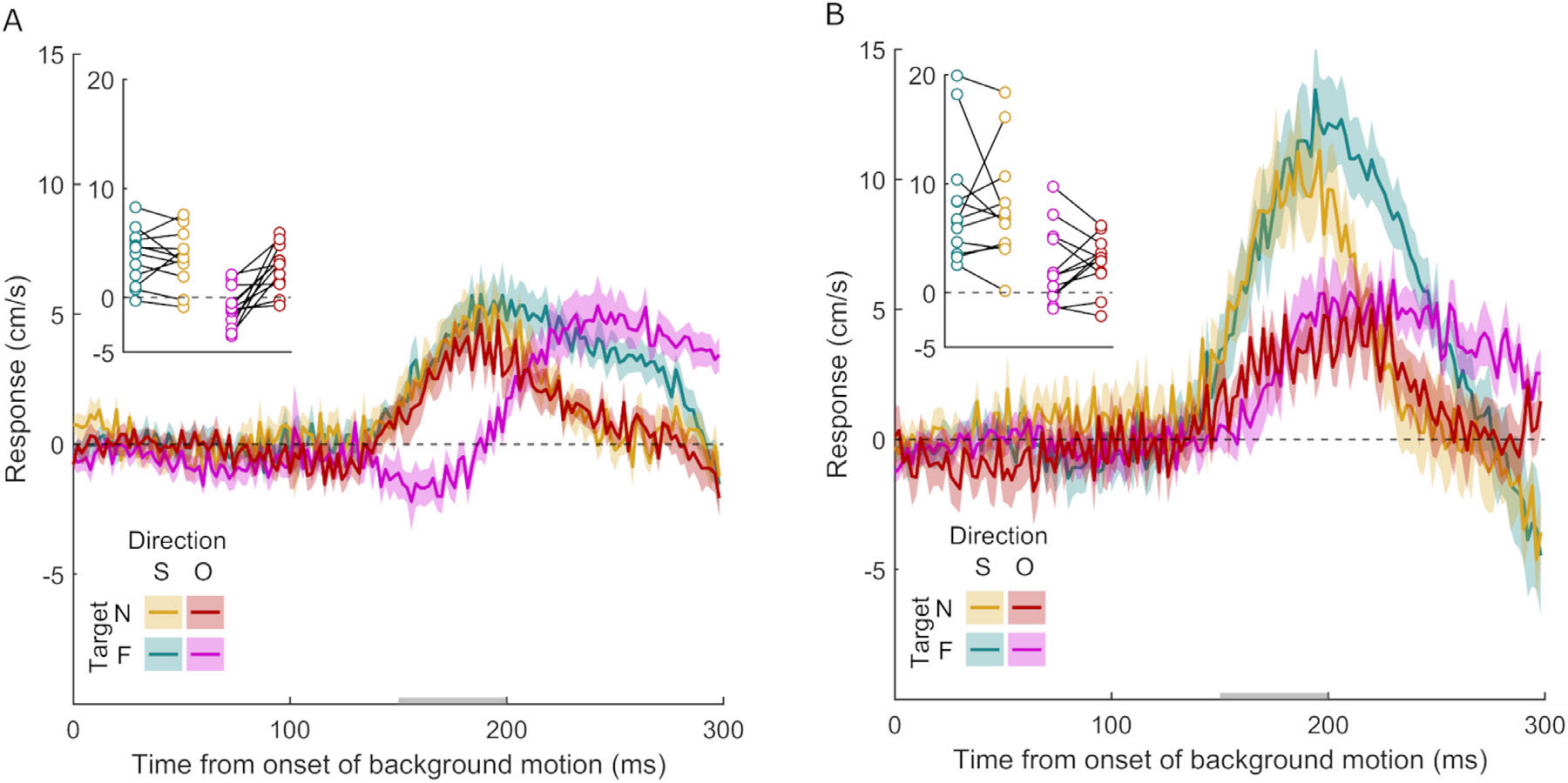
Time course of the response to background motion in Experiment 6b. (A) Experimental results. A positive response is a response in the direction of the background motion presented on the same screen as the target. This is relevant for the responses when the backgrounds on the two screens moved in opposite directions (red and purple curves for when the target was presented on the near and far screen, respectively). The gold and turquoise curves show the response when both backgrounds moved in the same direction, and the target was presented on the near and far screen, respectively. (B) Predictions for panel A based on a linear summation of the responses in Figure 7 (Experiment 6a). Further figure details are the same as Figure 2.

Experiment 6b examined the MFR when background motion was presented on both screens. Participants hit the screen 665 ± 26 ms after the target appeared and hit the target on 67  ± 3% of the 400 trials. Participants displayed the standard MFR when the background motion presented on the two screens moved in the same direction (gold and turquoise curves in Figure 8A). When the background dots on the two screens moved in opposite directions and the target was presented on the near screen, the hand deviated from its path in the direction of motion presented on the near screen (red curve in Figure 8A).

If the target was on the near screen, the motion on the far screen did not seem to matter: The gold and red curves in Figure 8A are very similar and are both quite similar to the thick turquoise curve in Figure 7, where background motion was exclusively presented on the near screen. Thus, although motion on the far screen did matter if there was no motion on the near screen (thin gold curve in Figure 7), when there was motion on the near screen, the influence of motion on the far screen appeared to be negligible. This was not the case when the target was on the far screen. If both screens moved in the same direction (turquoise curve in Figure 8A), the responses were initially similar to when the target was on the near screen (gold and red curves in Figure 8A). This is consistent with only background motion on the same screen as the target (or only background motion on the near screen) being relevant.

When the background dots on the two screens moved in opposite directions and the target was presented on the far screen (purple curve and data points in Figure 8A), the response was more complicated. There was no evidence for a consistent fast response of the hand away from its path (for the initial response at the target location, the 95% confidence interval is –2.1 to 0.25 cm/s). There does appear to be a consistent response in the direction of the far screen (i.e., the one with the target) after a delay.

This interpretation of the data is supported by a comparison with the sum of the effects found in Experiment 6a (Figure 8B). Such summing shows what one would expect to find if the two backgrounds’ influences on the MFR were independent, with no effect of the static background structures. The response to the same motion on both screens (gold and turquoise curves) is smaller in the data (Figure 8A) than one would expect from the summed responses from Figure 7 (Figure 8B). This is consistent with motion on the screen without the target being ignored when there is motion on both screens. When the target is on the near screen (red curves), the response is similar to the prediction based on summing the effects, but it is also quite similar to the response to background motion on the near screen alone (thick turquoise curve in Figure 7). When the target is on the far screen and the backgrounds move in opposite directions, the response is more difficult to interpret (purple curve in Figure 8A). The predictions reproduce the delay (purple curve in Figure 8B), but this is much more modest than in the actual data. It is possible that background motion on the near screen has an influence that is earlier than the effect of the far screen. This is the only case in which it is evident that the response is not simply dominated by the motion in the same plane as the target, although the response is still dominated by the direction of motion on the screen on which the action takes place (the responses are all mainly positive).

Overall, Experiment 6 shows that the depth at which the background moves relative to where the target moves is very relevant, so the region in which abrupt motion in the background leads to a response is defined in the three-dimensional space in which the hand moves rather than on the retina.

## General discussion

The MFR describes the response of the hand in the direction of sudden background motion when the hand is moving to a target. Several experimental characteristics of the MFR have already been documented that are inconsistent with the idea that the mechanism underlying this response is an estimation of self-motion based on an instantaneous global optic flow analysis (Zhang et al., 2019). Here, we find additional characteristics of the MFR that are also inconsistent with that idea. Specifically, we found that the response is not sensitive to the background's stability (Experiment 1) or static items in the background (Experiments 2, 3, and 6), but it is sensitive to where the motion takes place with respect to the movement endpoint (Experiments 3–6). Motion location matters in terms of the proximity to the interception zone, visual field, and depth. We propose a *movement stabilization mechanism* whereby the planned endpoint of one's action shifts in the direction of any motion in its vicinity. The shift is proportional to the motion signal, weighted according to the spatial relations between the background motion and planned endpoint, and the ongoing movement is then stabilized relative to this updated endpoint. Such a fast and simple movement stabilization mechanism is presumably an efficient way to quickly guide ongoing hand movements to their intended endpoints in dynamic situations.

Experiment 1 showed that the preceding stability of the environment is not considered. Rather, people appear to implicitly assume that the endpoint of their action is stable (Glennerster, Tcheang, Gilson, Fitzgibbon, & Parker, 2006). Experiment 2 showed that the MFR was still present when only one third of the background structures moved. Static structures appear to be ignored, which might seem strange because the participant's head is unlikely to be completely static and the participant is probably moving their eyes. Presumably, it is not whether a structure is moving across the retina that is critical but whether the motion changes abruptly. However, if so, it seems strange that giving the background dots limited lifetimes in Experiment 1 had such a small effect.

Even though 400 of the 600 dots were static in Experiment 2, while all 600 moved in the medium-stability condition of Experiment 1, the response was not reduced by a factor 3 (in accordance with the number of moving dots), let alone being further reduced by the presence of the static dots. These findings are not compatible with the idea that the MFR is elicited in response to estimating self-motion on the basis of the instantaneous optic flow because the motion signal from the background should indicate that the observer is static in these experiments. Indeed, only 3 moving dots among 597 static dots was sufficient to drive the MFR. Moreover, the predictions for Experiment 6b based on the data from Experiment 6a overestimated the magnitude of the MFR when the two screens moved in the same direction, suggesting that the static background structures in Experiment 6a did not reduce the magnitude of the MFR. These results show that not only is the MFR not eliminated by the presence of static structures but that static structures hardly influence the MFR. Since we know people are extremely apt at separating self-motion from object motion on the basis of optic flow (Warren & Rushton, 2007, 2008), this provides definitive evidence against the MFR being guided by a mechanism that estimates self-motion based on the instantaneous optic flow. It is consistent with a direct response to local abrupt motion.

The movement stabilization mechanism specifies that *any* sudden motion in a given region is adequate to elicit a response. It is an automatic response that simply updates the planned endpoint of the movement to match the motion in the vicinity. It does not involve an explicit estimation of self-motion and is therefore compatible with the MFR being more prominent than postural responses to background motion (Zhang et al., 2018) and to it not being found after illusory self-motion induced by vestibular stimulation (Zhang et al., 2019), as well as with the finding that the presence of static structures does not influence the MFR. It can also explain the experimental characteristics of the MFR we report here: The preceding stability of the environment and the presence of static background structures are not considered. Indeed, any sudden motion signal results in the observer stabilizing their movement relative to an endpoint location that is updated by such motion.

The movement stabilization mechanism accounts for findings showing that the MFR is a well-localized response (Abekawa & Gomi, 2010; Brenner & Smeets, 2015). In line with the proposal that the movement is stabilized relative to the planned endpoint of one's action, background motion at the interception zone was particularly effective in driving the MFR (Experiment 3). Experiment 6 also provides support for the idea that the hand moves with respect to the interception zone by showing that depth is considered. However, the response to three dots moving near the target in Experiment 3, the response to motion in the lower part of the screen in Experiment 4, and the response to motion at a different depth than the target in Experiment 6 show that the MFR does not only depend on motion precisely at the anticipated point of interception. There is some range near the anticipated interception point within which any abrupt motion gives rise to the MFR. The movement stabilization mechanism thus shifts the planned endpoint according to a motion signal that appears to be weighted according to the spatial relations between background motion and planned endpoint. The preferential processing of motion near the planned endpoint of one's action presumably serves to facilitate a fast, direct estimate of the stabilization required for one to successfully reach one's target.

The experimental characteristics of the MFR that we found in these experiments are also inconsistent with other mechanisms that have been proposed to underlie the MFR. The fact that the response was stronger when the three points that moved were near the interception point than when they were near the target and that not only motion along the target's path drives the MFR shows that the MFR is not the result of background motion near the target being incorrectly attributed to the target (Zhang et al., 2019). Our finding that the MFR still emerged when background motion was presented exclusively in the upper half of the screen clearly refutes the suggestion that the MFR is the result of background motion influencing the apparent motion of the hand (Grierson & Elliot, 2009) because in that case, there is no background motion in the vicinity of the hand.

Our finding in Experiment 5 that motion below the target's path is more influential than that above the target's path is somewhat inconsistent with the findings of Brenner and Smeets (2015, Experiment 3), which suggested that regions above the interception location were very influential. A possible explanation for this is that their study used large squares at fixed positions, whereas here we used many small randomly distributed dots. When using large squares, the actual motion signal is probably provided by the edges of the squares, and the closest edge to the interception point is always that between the square behind the interception point and the one above it (only horizontal edges are relevant because the background moved vertically in that study). That may be why the strongest MFR was found when that edge moved, which happened when either the square behind the interception point or the one above it moved. Using random dot positions in the current study has the advantage that each moving item has an independent, spatially distinct influence on the MFR such that we can be more confident about the spatial bias that is revealed.

The depth effects could partly be related to the location of participants’ gaze (and accommodation). Although we did not measure eye movements in these experiments, it is reasonable to assume that participants were fixating the target or interception zone, both of which were always on the same screen. This might contribute to the MFR being larger when the background moved at the same distance as the target in Experiment 6, because the background on the un-fixated depth plane is likely to be less well fused and its retinal image is likely to be slightly blurred. This is obviously also the case in daily life and might at least partially account for the selectivity in depth. Other effects are less likely to be related to gaze. Considering the relative sizes of the target and interception zone, we expect participants to have been pursuing the target with their eyes (Brenner & Smeets, 2015), but of course, we cannot be sure that they were doing so rather than fixating the interception zone. It is therefore premature to conclude that the results of Experiment 3 show that it is motion at the interception point rather than at the point of fixation that is critical. For many movements in daily life, the two will be the same.

In general, background motion near the interception zone appeared to dominate the MFR. However, there was also a tendency for motion in regions closer to the moving hand to be considered more important than other regions. We observed such a bias in Experiments 3 and 4 for background motion below the target. We also observed this in Experiment 6b, where motion of the background just above the hand influenced the response to background motion in the opposite direction near the target (purple curve in Figure 8B), whereas motion of the background on the far screen did not influence the response to background motion on the screen near the target (gold and red curves are extremely similar in Figure 8B).

## Conclusions

When making goal-directed movements toward a target, our hand is pulled in the direction of background motion. We propose that a *movement stabilization mechanism* that serves to guide the hand to the movement endpoint explains this MFR. Our results are consistent with the proposal that any sudden motion near the planned endpoint updates the position of that planned endpoint such that the hand follows the direction of background motion in an attempt to stabilize the ongoing movement.
